# A quasi-hyperbolic discounting approach to smoking behavior

**DOI:** 10.1186/s13561-014-0005-7

**Published:** 2014-06-17

**Authors:** Takanori Ida

**Affiliations:** 1 Graduate School of Economics, Kyoto University Yoshida, Sakyo 6068501, Kyoto, Japan

**Keywords:** Smoking, Cigarette dependence, Time preference, Present bias, D81; D91; I12

## Abstract

Addiction has attracted considerable attention in health and behavioral economics, and economists have attempted to understand addiction from the viewpoint of decision making over time. This paper investigates whether two time preference parameters can successfully predict smoking status, including cigarette dependence. Both the present bias and the constant time preference parameters account for smoking behavior status and cigarette dependence.

## Background

Addiction has attracted considerable attention in health and behavioral economics, and economists have attempted to understand addiction from the viewpoint of decision making over time (Chaloupka and Warner [1]). This viewpoint is relevant because consumers believe that although an addictive product such as tobacco may increase their current satisfaction, it actually decreases their future utility by damaging their health^a^.

The purpose of this study is to investigate smoking status, including cigarette dependence (the most common form of addiction), using the quasi-hyperbolic discounting approach proposed by Laibson [2]. When one compares the current utility of smoking (i.e., temporary stress relief) with the future utility of non-smoking (long-term good health), individuals that have a higher time preference rate tend to attach larger importance to the former compared with the latter and are thus more likely to smoke (and moreover be heavily addicted). Further, if an individual has a present bias, namely his or her current utility is especially high compared with future utility, he or she is more likely to start smoking and to fail to quit smoking many times despite acknowledging the health benefits of not smoking. In this sense, some smokers neither recognize the true difficulty of quitting nor search for self-control devices to help themselves quit. Thus, government policy should consider not only the externalities imposed by smokers on others but also the internalities imposed by smokers on themselves (Gruber and Koszegi [3]; Kan [4]).

This study first tests the likelihood that the stationarity axioms, which are required according to discounted utility theory, are violated. It then investigates whether these parameters can successfully predict smoking status, including cigarette dependence, based on a quasi-hyperbolic (*β-δ*) discount function, where the parameter *β* denotes present bias and *δ* is the standard exponential (constant) discount factor.

As Doyle [5] pointed out, quasi-hyperbolic discounting has rarely been used in psychological research, though it has been used extensively by economists attempting to preserve the exponential model. Nevertheless, quasi-hyperbolic discounting lends itself to convenient testing against normative exponential discounting by testing whether the present bias value (*β*) is significantly less than 1. Yet, it has rarely been tested in the literature. The only exception is Van de Ven and Weale [6], who noted *β* ranging from 0.296 to 0.825. The contribution of my paper is that I explicitly measure the *β* values on the basis of quasi-hyperbolic discounting and quantitatively relate them to smoking behavior.

This paper’s main contributions to the body of knowledge can be summarized as follows. First, by analyzing whether quasi-hyperbolic discounting parameters are associated with smoking, I find that both the present bias and the constant time preference parameters account for smoking behavior very well. Elasticity, which measures how changing one economic variable affects others, also helps quantify this relationship. The analysis shows that a 1% increase in the present bias parameter significantly increases smoking probability by 0.42%, while a 1% increase in the constant time preference parameter increases smoking probability by 0.68%.

Second, I investigate how these parameters elucidate cigarette dependence and find that both the present bias and the constant time preference parameters also account for cigarette dependence very well. The analysis shows that a 1% increase in the present bias parameter decreases the proportion of low nicotine-dependent smokers by 0.43% but increases that of highly nicotine-dependent smokers by 0.27%. Furthermore, a 1% increase in the constant time preference parameter decreases the proportion of low nicotine-dependent smokers by 1.21% but increases that of highly nicotine-dependent smokers by 0.84%. Thus, I can conclude that quasi-hyperbolic discounting parameters function as good predictors of smoking status.

The remainder of this paper is organized as follows. Section II describes the survey method of sampling data and the research strategy. Section II.1 classifies the samples as time consistent and time inconsistent. Section II.2 explains the measurement of preference parameters. Section II.3 presents the estimation model. Section III investigates four hypotheses. Section IV discusses the results, and Section V provides concluding remarks.

## Methods

In July 2008, we surveyed 494 Japanese adults registered with a consumer monitoring investigative company. Our survey was conducted before the radical increase of tobacco in October 2010. We here note that the results may be affected by the small sample properties. The sample was adjusted to reflect Japanese demographics in terms of gender, average age, and geographical features. A total of 150 Japanese Yen (JPY) (1.5 US$, given 100 JPY = 1 US$) was paid to respondents to the basic survey questionnaire and 500 JPY (5 US$) was paid to respondents to the discrete choice experiments questionnaire described below. Respondents who answered in an unrealistically short period of time were excluded from the final sample.

Of the 494 participants sampled, 241 (48.8%) were non-smokers (including 59 ex-smokers defined as individuals who had quit smoking and had not smoked for at least one year). Since ex-smokers may be different from those sample participants that had never smoked, I separated ex-smokers from non-smokers in the analysis. In terms of demographics, the proportion of smokers (non-smokers) who were women was 36.4% (51.5%). The average ages of smokers and non-smokers were 40.5 and 38.1 years, respectively, while 46.2% of smokers and 69.7% of non-smokers were university or junior college graduates. Annual household incomes were 5.9 million JPY (59,000 US$) for smokers and 6.3 million JPY (63,000 US$) for non-smokers. The basic statistics are summarized in Table 1.

**Table 1:** Basic demographics

	**Sample no.**	**Time inconsistent ratio**	**Female ratio**	**Average age**	**Year of education**	**Average household income (JPY)**
Non-smoker	241	0.30	0.52	38.14	14.91	6.3 M
			(0.50)	(12.63)	(2.02)	(3.6 M)
Never-smoker	182	0.30	0.57	35.26	15.00	6.1 M
			(0.50)	(11.25)	(1.97)	(3.4 M)
Ex-smoker	59	0.31	0.36	47.02	14.63	7.2 M
			(0.48)	(12.63)	(2.16)	(3.9 M)
Smoker	253	0.35	0.36	40.48	13.83	5.9 M
			(0.48)	(11.88)	(2.20)	(3.7 M)
L-Smoker	97	0.33	0.48	38.30	14.08	5.7 M
			(0.50)	(11.73)	(2.10)	(3.49 M)
M-Smoker	111	0.32	0.32	40.86	13.76	6.3 M
			(0.47)	(12.45)	(2.18)	(3.79 M)
H-Smoker	45	0.47	0.22	44.22	13.49	5.5 M
			(0.42)	(9.79)	(2.41)	(3.62 M)

Note 1: The values in parentheses denote the standard errors.

I defined cigarette dependence as follows. Fagerström [7] recognizes that although nicotine is the most important addictive component in tobacco smoke, it is probably not the only substance involved in the development of tobacco dependence. In this light, this paper replaces the Fagerström Test for Nicotine Dependence (FTND) with the Fagerström Test for Cigarette Dependence (FTCD). FTCD comprises the following six questions (Heatherton et al. [8]):

1. How soon after you wake up do you smoke your first cigarette? (1) Within 5 minutes (3 points), (2) 6–30 minutes (2 points), (3) 31–60 minutes (1 point), (4) After 60 minutes (0 points)

2. Do you find it difficult to refrain from smoking in places where it is forbidden, e.g., in church, at the library, at the cinema, etc.? (1) Yes (1 point), (2) No (0 points)

3. Which cigarette would you hate most to give up? (1) The first one in the morning (1 point), (2) All others (0 points)

4. How many cigarettes do you smoke a day? (1) 10 or less (0 points), (2) 11–20 (1 point), (3) 21–30 (2 points), (4) more than 30 (3 points)

5. Do you smoke more frequently during the first hours after waking than during the rest of the day? (1) Yes (1 point), (2) No (0 points)

6. Do you smoke even if you are so ill that you are in bed most of the day? (1) Yes (1 point), (2) No (0 points)

By aggregating the responses, we defined respondents with 0 to 3 points as low cigarette dependence (L-smokers), 4 to 6 points as moderate cigarette dependence (M-smokers), and 7 and over as high cigarette dependence (H-smokers). Altogether, 38.3% of respondents were L-smokers, 43.8% M-smokers, and 17.8% H-smokers. The proportions of female and university graduates were highest for L-smokers, average age was highest for H-smokers, and average income level was highest for M-smokers.

The following four hypotheses were tested in this paper:

*Constant time preference* and smoking probability:

The higher the time preference rate, the higher is smoking probability.

*Present bias* and smoking probability:

The higher the present bias effect, the higher is smoking probability.

*Constant time preference* and cigarette dependence:

The higher the time preference rate, the higher is cigarette dependence.

*Present bias* and cigarette dependence:

The higher the present bias effect, the higher is cigarette dependence.

**Figure 1:**
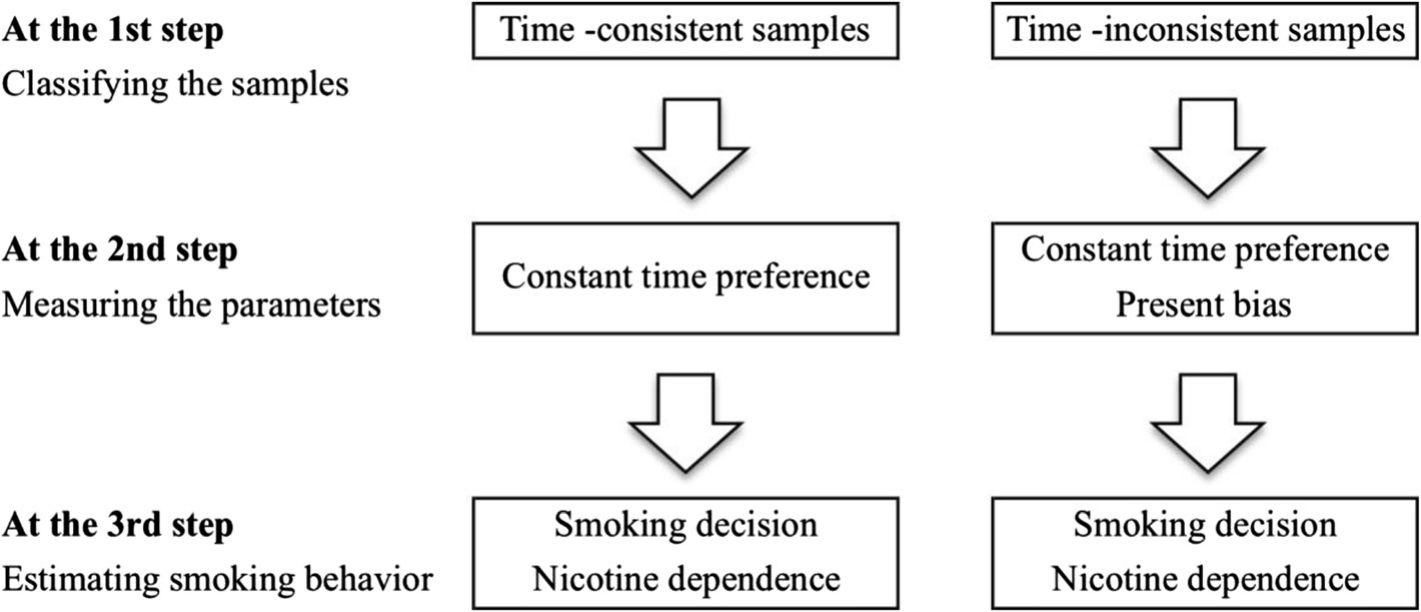
Research strategy.

The research strategy adopted herein to test these four hypotheses was composed of the following three steps. First, I conducted an experimental survey to assess whether smokers displayed exponentially discounted utility anomalies. Then, I classified the whole sample into time-consistent and time-inconsistent subsamples. Second, I used the analysis to measure the present bias parameter for the time-inconsistent sample and the constant time preference parameter for both the time-consistent and the time-inconsistent samples. Third, I investigated the influence of these factors on the probability of smoking and of cigarette dependence by using the ordered probit model (structural equation) with a binomial probit model (selection equation). Figure 1 depicts this research strategy.

### STEP 1: classifying respondents as time consistent or time inconsistent

The standard theory of decision making over time is based on the exponentially discounted utility model, whose key assumption is a stationarity axiom. This axiom implies that if and only if the utility of 100,000 JPY in the present is preferred to the utility of 150,000 JPY after one year, then the utility of 100,000 JPY after 10 years is preferred to the utility of 150,000 JPY after 11 years, because the implicit discount factor should be the same in both cases.

We assume an intertemporal consumption decision with consumption in the current year (*C*_0_) and in the following year (*C*_1_). This representation makes clear that the marginal rate of substitution (MRS) at an agent’s chosen consumption bundle (or the observed gross rate of time preference) depends on two factors: constant pure time preference (*δ*) and diminishing marginal utility (*U*′(*C*_0_)/*U*′(*C*_1_)) because

(1) $$\mathrm{MRS}=-{\frac{dC_{1}}{dC_{0}}|}_{\mathrm{dU}0=0}=\frac{U^{\prime }\left(C_{0}\right)}{U^{\prime }\left(C_{1}\right)}\times \left(1+\delta \right)$$

Frederick et al. [9] pointed out that economists are not comfortable using the term “time preference” to include the effects of differential marginal utility arising from unequal consumption levels between time periods. In that sense, economists tend to focus on the exponentiality of the pure rate of time preference^b^.

Assuming here that *X* and *Y* denote payoffs (*X < Y*) and *t* and *s* denote time delay (*t < s*), the axiom is more formally defined as follows:

(2) $$\left(X,t\right)\geq \left(Y,s\right)\mathrm{and}\left(X,t+\varepsilon \right)\geq \left(Y,s+\varepsilon \right).$$

Note that *ε* is a positive constant.

At this point, the exponentially discounted utility model gives *U(X)/(1 + r)*^*t*^ ≥ *U(Y)/(1 + r)*^*s*^ for *t* and *s*. However, the discounted utility anomaly of a present-smaller reward being excessively preferred to a delayed-larger reward indicates the following inconsistent preference orders:

(3) $$\left(X,t\right)\geq \left(Y,s\right)\mathrm{and}\left(X,t+\varepsilon \right)\leq \left(Y,s+\varepsilon \right).$$

This anomaly is called *time inconsistency,* which is sometimes referred to as *decreasing impatience* (Strotz [10]; Prelec [11]). For example, Takahashi [12] demonstrated that time inconsistency is proportional to the Arrow–Pratt concavity of nonlinear time perception (i.e., decreasing impatience).

I asked respondents two hypothetical questions in order to investigate the discounted utility anomaly:

Question 1

 Alternative 1: Receive 100,000 JPY (1,000 US$) *immediately*.

 Alternative 2: Receive 150,000 JPY (1,500 US$) *after T years*.

 What *T* makes the two alternatives equivalent?

Question 2

 Alternative 1: Receive 100,000 JPY (1,000 US$) *after one year*.

 Alternative 2: Receive 150,000 JPY (1,500 US$) *after S years*.

 What *S* makes the two alternatives equivalent?

Based on the exponentially discounted utility model, when the utility of 100,000 JPY in the present equals the utility of 150,000 JPY *after T years*, I obtain the following equation:

(4) $$\mathrm{Utility}\;\mathrm{of}\;100,000\;\mathrm{JPY}=\mathrm{Utility}\;\mathrm{of}\;150,000\;\mathrm{JPY}/{\left(1+r\right)}^{T}.$$

Note that *r* denotes the annual time preference rate.

Further, when the utility of 100,000 JPY *after one year* equals the utility of 150,000 JPY *after S years*, I obtain the following equation:

(5) $$\mathrm{Utility}\;\mathrm{of}\;100,000\;\mathrm{JPY}/\left(1+q\right)=\mathrm{Utility}\;\mathrm{of}\;150,000\;\mathrm{JPY}/{\left(1+q\right)}^{S}.$$

If the time preference rate is constant (*r = q)*, as the exponentially discounted utility model assumes, then *T/(S – 1)* = 1 holds. However, the discounted utility anomaly *T/(S – 1) < 1* is frequently observed, so the time preference rate decreases for time delay (*r > q*). The main reason for this is the *present bias effect*, wherein people tend to place disproportionally more emphasis on an immediate reward as opposed to a delayed one (Frederick, et al. [9]). For example, in Question 1, because Alternative 1 consists of an immediate reward, Alternative 2 requires that *T* be a relatively small figure (e.g., one year). By contrast, in Question 2, because Alternative 1 consists of a one-year-delayed reward, Alternative 2 requires that *S* be a large figure (e.g., three years). The time consistency index is defined as *T/(S – 1). T/(S – 1)* = 1 indicates perfect consistency, while *T/(S – 1)* = 0 indicates perfect inconsistency. It follows that *T/(S – 1) = 0.5* for the example above. In this way, I classify the samples as time consistent if *T/(S – 1)* = 1 and time inconsistent otherwise. One limitation is that I use only two questions to address the discounted utility anomaly. It would be desirable in future research to present a greater number of questions and classify the degree of anomaly into multiple levels.

Table 1 (right row) summarizes the proportions of the samples that are time inconsistent. The proportions are 0.299 for non-smokers and 0.352 for smokers, indicating that the behaviors of non-smokers are more consistent with the discounted utility hypothesis than those of smokers. For smokers, the proportions are 0.330 for L-smokers, 0.324 for M-smokers, and 0.440 for H-smokers, indicating that high cigarette dependence is associated with a less consistent time preference. Moreover, the proportions are 0.297 for those that had never smoked and 0.305 for ex-smokers, showing that the tendency is similar for these groups.

### STEP 2: estimating the time and risk preference parameters

The methodology of measuring the preference parameters in this study follows the approach presented by Ida and Goto [13], who surveyed 692 respondents to simultaneously assess time and risk preferences by using the choice-based DCE model, finding that smokers are more impatient and more risk-prone than non-smokers. However, they failed to differentiate present bias and the constant time preference rate. To address this shortcoming, a new survey was conducted in the present study by adding the STEP 1 questionnaire in August 2008 (Ida [14]). Respondents were classified as time consistent or time inconsistent based on the exploratory open-ended matching method. I then separately measured the constant time preference and present bias parameters (along with the risk preference coefficients) at the individual level in STEP 2.

Based on the foregoing, the DCE model was used herein to simultaneously measure the time and risk preferences of the 494 respondents, given that an alternative is a profile composed of attributes. In Alternative 1, the baseline alternative, the levels of the reward, probability, and delay were fixed across profiles, whereas these attributes varied across profiles in Alternative 2. After conducting several pretests, I thus determined the alternatives, attributes, and levels presented in Table 2.

**Table 2:** Attributes and levels

**Alternative 1**				
Attributes	Levels			
Reward:	100,000 JPY			
	(1,000 US$)			
Winning probability:	100%			
Time delay:	None			
**Alternative 2**				
Attributes	Levels			
Reward:	150,000 JPY	200,000 JPY	250,000 JPY	300,000 JPY
	(1,5000 US$)	(2,000 US$)	(2,500 US$)	(3,000 US$)
Winning probability:	40%	60%	80%	90%
Time delay:	1 month	6 months	1 year	5 years

Because the number of profiles would become unmanageable if all possible combinations were considered, an orthogonal planning method was adopted. The 16 questions were divided into two versions, and respondents were asked to answer either version. Therefore, I posed eight questions to each respondent.

Next, in the quasi-hyperbolic discount function (Laibson [2]), lifetime utility from present period 0 onwards is given by

(6) $$u_{0}+\exp\left(\beta \right)\sum_{t=1}^{T}\exp\left(-\mathrm{\delta t}\right)u_{t}$$

where *μ*_*t*_ is periodic utility, the parameter *β* denotes present bias, and *δ* is the standard exponential discount factor.

#### Time-consistent samples

Let the utility of alternative *i* be *V*_*i*_ (reward_i_, probability_i_, timedelay_i_). The exponentially discounted and expected utility model is assumed for time-consistent samples to derive the functional form of *V*_*i*_ as follows:

(7) $$\begin{array}{l} V_{i}\left({\mathrm{reward}}_{i},{\mathrm{probability}}_{i},{\mathrm{timedelay}}_{i}\right)\\ =\exp\left(–\delta *{\mathrm{timedelay}}_{i}\right)*{\mathrm{probability}}_{i}*\mathrm{utility}\left({\mathrm{reward}}_{i}\right), \end{array}$$

where *δ* denotes the constant rate of time preference.

I specify the functional form of utility as the *γ*-th power of reward. Such a utility function is called the constant relatively risk-averse form, where the coefficient of relative risk aversion is denoted by 1-*γ*. By taking the logarithm of both sides, I obtain

(8) $$\begin{array}{l} \ln\;V_{i}\left({\mathrm{reward}}_{i},{\mathrm{probability}}_{i},{\mathrm{timedelay}}_{i}\right)\\ =\ln\;{\mathrm{probability}}_{i}–\delta *{\mathrm{timedelay}}_{i}+\gamma *\ln\;{\mathrm{reward}}_{i} \end{array}$$

#### Time-inconsistent samples

The following quasi-hyperbolically discounted and expected utility model is assumed for the time-inconsistent sample:

(9) $$\begin{array}{l} V_{i}\left({\mathrm{reward}}_{i},{\mathrm{probability}}_{i},{\mathrm{timedelay}}_{i}\right)\\ =\exp\left(\beta *\left[{\mathrm{timedelay}}_{i}\right]\right)*\exp\left(\delta *{\mathrm{timedelay}}_{i}\right)*\mathrm{probabilit}y_{i}*\mathrm{utility}\left({\mathrm{reward}}_{i}\right), \end{array}$$

where *β* denotes present bias, 1[timedelay_i_] is an index function for a delayed reward in alternative 2, and *δ* is the constant rate of time preference.

Again, by taking logarithms and assuming a constant relative risk-averse form (*γ*), I obtain

(10) $$\begin{array}{l} \ln V_{i}\left({\mathrm{reward}}_{i},{\mathrm{probability}}_{i},{\mathrm{timedelay}}_{i}\right)\\ =\ln\;{\mathrm{probability}}_{i}+\beta *1\left[{\mathrm{timedelay}}_{i}\right]–\delta *{\mathrm{timedelay}}_{i}+\gamma *\ln\;{\mathrm{reward}}_{i}. \end{array}$$

Thus, *δ* is estimated for both the time-consistent and the time-inconsistent samples, while *β* is estimated only for the time-inconsistent sample.

Finally, conditional logit (CL) models, which assume the independent and identical distribution (IID) of random terms, have been widely used in previous studies. Recently, the most appropriate scheme to adopt has been a random parameters (or mixed) logit (RPL) model, which can accommodate differences in the variance of random coefficients. Such models are flexible enough to overcome the limitations of CL models by allowing random taste variation, unrestricted substitution patterns, and the correlation of random terms by choice situation. Fiebig et al. [15] argued, furthermore, that much of the heterogeneity in attribute coefficients is accounted for by scale heterogeneity and thus that the scale of their error term is allowed to be larger for some consumers than others by fixing the attributes’ coefficients. A generalized mixed (or scaled random parameters) logit model that includes a free-scale parameter to be estimated was adopted in this work. See Appendix for the technical details.

It is assumed here that the random parameters follow a normal distribution. One can demonstrate variety in the parameters at the individual level by using the maximum simulated likelihood method for estimation with 200 Halton draws. Further, as respondents answered eight questions as part of the DCE analysis, the resultant data form a panel that offers the option of applying a standard random effect estimation. Hence, the estimator of the conditional means of the random parameters can be calculated at the individual level (denoted by subscript *n*), *β*_*n*_, *δ*_*n*_, and *γ*_*n*_. These individual-level preference parameters are used as explanatory variables in the STEP 3 estimation.

Table 3 summarizes the measurement results. First, the basic fact that smokers are more impatient than non-smokers is observed: the measured *constant* monthly time preference rates (*δ*_*n*_) are 6.8% for smokers and 5.6% for non-smokers. Specifically, these rates are 5.9% for L-smokers, 8.0% for M-smokers, and 8.6% for H-smokers, indicating that heavier smokers are more impatient.

**Table 3:** Impatience, present bias, and risk parameters

				**Random parameters**		
	**Log likelihood**	**Scale parameter**		**δ (constant time preference)**	**Exp(β) (present bias)**	**1-γ (relative risk aversion)**
Non-smoker	-587.7	0.3299 (0.1197)	Mean	-0.0556 (0.0098)***	0.4578 (0.1909)***	-0.2283 (0.2065)
(N = 241)			S.D.	0.0346 (0.0101)***	0.0617 (0.0790)	0.4064 (0.2101)*
Never-smoker	-440.1	0.2318 (0.1652)	Mean	-0.0542 (0.0131)***	0.4207 (0.1956)**	-0.2172 (0.2438)
(N = 182)			S.D.	0.0261 (0.0120)**	0.0875 (0.0976)	0.0207 (0.7300)
Ex-smoker	-140.1	0.2718 (0.2341)	Mean	-0.0700 (0.0253)***	0.5365 (0.5339)	-0.3697 (0.4689)
(N = 59)			S.D.	0.2077 (0.1219)*	0.0562 (0.0316)*	0.8366 (0.4635)*
Smoker	-57105	0.3575 (0.1073)***	Mean	-0.0683 (0.0133)***	0.3619 (0.1137)***	-0.3658 (0.2186)
(N = 253)			S.D.	0.0421 (0.0127)***	0.0276 (0.1055)	0.6265 (0.2108)***
L-smoker	-226.2	0.3900 (0.2694)	Mean	-0.0587 (0.0211)***	0.2685 (0.1076)**	-0.4843 (0.3617)
(N = 97)			S.D.	0.0341 (0.0192)**	0.0072 (0.1253)	0.6083 (0.3096)**
M-smoker	-255.6	0.3575 (0.1461)**	Mean	-0.0802 (0.0226)***	0.5677 (0.5316)	-0.2035 (0.3468)
(N = 111)			S.D.	0.0465 (0.0188)**	0.1129 (0.3377)	0.7142 (0.3870)*
H-smoker	-93.8	0.3575 (0.1908)*	Mean	-0.0855 (0.0365)**	0.2876 (0.1597)*	-0.5267 (0.5171)
(N = 45)			S.D.	0.0639 (0.0334)*	0.0305 (0.1605)	0.3395 (1.1399)

Note 1: The values in parentheses denote the standard errors of MEAN and S.D. estimates for the random parameters. ***1% significant level (p < 0.01), **5% significant level (p < 0.05), *10% significant level (p < 0.1).

Note 2: δ (constant time preference) and 1-γ (relative risk aversion) are estimated for both time-consistent and time-inconsistent samples, while β (present bias) is estimated only for the time-inconsistent samples.

Note 3: the coefficient is normalized to one for the variable 1n probability, which appear as separate shift parameters (according to the level of probability).

Note that the measured time preference rates are very high compared with those presented in the economic literature, partly because I estimated the preferences using a hypothetical survey and because the absent income constraints framework leads to biased responses. Further, the discount factor is a function of the time horizon, which I partly address by the present bias effect, and this is conspicuous when I consider intertemporal choices within one year. Fredrick et al. [9] also pointed out the huge variability in discount rate estimation (from negative to infinity).

However, simultaneously measuring the constant time preference (*δ*_*n*_) and present bias parameters (exp(*β*_*n*_)) leads to some unexpected results. Although smokers (0.36) have higher present bias than non-smokers (0.46), M-smokers (0.57) have lower present bias than L-smokers (0.27) and H-smokers (0.29). This finding may mean that M-smokers suffer the least from present bias.

Another counterintuitive result is that the measured risk values (1-*γ*_*n*_) are negative. However, none of the coefficients of relative risk aversion is statistically significant. This finding may occur because the functional forms assumed herein are so specific that any unobserved interdependencies among the parameters are insufficiently addressed. Indeed, although many studies have investigated the relationship between smoking and attitudes toward risk, this issue remains inconclusive (Mitchell [16]; Reynolds et al. [17]; Ohmura et al. [18]).

### STEP 3: estimating smoking decision and cigarette dependency

The smoking decision can be divided into two steps: (i) the decision to start smoking and (ii) the degree of cigarette dependence. This two-step decision is considered to be an ordered probit model (in which cigarette dependence is classified into three groups depending on FTCD scores) with a binomial probit model (in which smoking is denoted by 1 and non-smoking by 0).

The selection equation is a binominal probit model written as follows (McKelvey, and Zavoina [19]):

(11) $$\begin{array}{l} d_{n}^{*}=a^{'}X_{n}+b*\left(1-\exp\left(\beta_{n}\right)\right)+c*\delta_{n}+d*\left(1-\gamma_{n}\right)+u_{n},\\ d_{n}=1\;\mathrm{if}\;d_{n}^{*}>0\;\mathrm{and}\;0\;\mathrm{otherwise}. \end{array}$$

The structural equation is an ordered probit model written as follows:

(12) $$\begin{array}{l} y_{n}^{*}=a^{'}X_{n}+b*\left(1-\exp\left(\beta_{n}\right)\right)+c*\delta_{n}+d*\left(1-\gamma_{n}\right)+\varepsilon_{n},\\ \;\mathrm{where}\;\varepsilon_{n}∼\Phi \left(\varepsilon_{n}|\theta \right),\;E\left[\varepsilon_{n}\right]=0,\;\mathrm{Var}[\varepsilon_{n}]=1,\\ y_{n}=0\;\mathrm{if}\;{y_{n}}^{*}\leq \;0;\\ \;=1\;\mathrm{if}\;0\;\leq \;{y_{n}}^{*}\leq \;\mu ;\\ \;=2\;\mathrm{if}\;\mu \;\leq \;{y_{n}}^{*}. \end{array}$$

As definitions of variables, *d*_*n*_ denotes the decision to start smoking, *y*_*n*_ denotes the degree of cigarette dependence, and *X*_*n*_ includes constant time preference, present bias, gender, age, age squared, the year of education, household income, and relative risk aversion. The system [*y*_*n*_, *X*_*n*_] is observable if and only if *d*_*n*_ = 1 holds. Selectivity matters if *ρ* is not equal to zero:

(13) $$\left[\varepsilon_{n},u_{n}\right]∼N\left[0,0,1,1,\rho \right].$$

The elasticities in the ordered probit model (w.r.t. *Χ*_*n*_) can be calculated as the effects of changes in the covariates on each range of probability:

(14) $$\begin{array}{l} \left[\partial \mathrm{Pr}\mathrm{ob}\left[y_{n}=j\right]/\partial X_{n}]\times [X_{n}/\mathrm{Pr}\mathrm{ob}[y_{n}=j]\right]\\ =\left[\phi \left(\mu_{j-1}-y_{j}^{*}\right)-\phi \left(\mu_{j}-y_{j}^{*}\right)\right]\times b\times \left[X_{n}/\mathrm{Pr}\mathrm{ob}\left[y_{n}=j\right]\right] \end{array}$$

where *j = 0, 1,* and *2* and *ϕ* denotes normal density.

Elasticities are measured around the mean values.

The full information maximum likelihood method is then used to estimate the parameters, including *ρ*. This method reduces to the limited information maximum likelihood method if *ρ=*0 holds. The explained variables are given as follows. In the binomial model, the dummy variable is 1 for smoking and 0 for non-smoking, while in the ordered probit model, the variable for cigarette dependence ranges from 0 (low) to 2 (high).

The explanatory variables are the present bias effect, the rate of time preference, and the rate of risk preference. Note that the present bias effect is measured as 1 − exp(*β*_*n*_) rather than exp(*β*_*n*_) at this point. The individual characteristic variables are dummy variables for gender (*GENDER* = 0 for male), age (*AGE*), age squared (*AGESQ*), year of education (*EDUCATION*), and annual household income (*INCOME*, million JPY).

## Results

The estimation results are shown in Table 4 with the results of the selection equation model. Regarding the two key parameters, both constant time preference and present bias are significantly associated with smoking probability, whereas the risk preference rate has a significant influence. The gender and school history dummies are negatively associated with smoking probability, while age is reverse U-shaped (with the peak around 55 years old). Finally, annual household income does not influence smoking probability.

**Table 4:** Estimation results

**Sample no**		**435**	
**Log likelihood**		**-516.2776**	
	**Coefficient**	**S.E.**	
Selection equation			
δ (constant time preference)	16.2753	3.7047	***
1-exp(β) (present bias)	1.9808	0.8804	**
Gender	-0.46059	0.13383	***
Age	0.07791	0.02441	***
Age squared	-0.00071	0.0003	*
Education (Years)	-0.13476	0.02857	***
Income (M JPY)	-0.00447	0.02102	
Relative risk aversion (1-γ)	-0.8784	0.26358	***
Structual equation			
δ (constant time preference)	21.8066	4.5830	***
1-exp(β) (present bias)	2.1157	0.7006	***
Gender	-0.74757	0.21399	***
Age	0.05826	0.02403	**
Age squared	-0.00043	0.00028	
Education (Years)	-0.10187	0.05602	*
Income (M JPY)	-0.00331	0.0176	
Relative risk aversion (1-γ)	-0.47637	0.27835	*
Threshold parameter	2.35304	0.12287	***

Note: ***1% significant level (p < 0.01), **5% significant level (p < 0.05), *10% significant level (p < 0.1).

Then, I discuss the results of the structural equation model. I conducted the full information maximum likelihood estimation, but could not reject the null hypothesis (i.e., *ρ* = 0. Similar to the results above, both constant time preference and present bias are significantly associated with cigarette dependence, while the risk preference rate negatively influences cigarette dependence. The gender and school history dummies are negatively associated with cigarette dependence, while age is reverse U-shaped (with the peak around 68 years old). Finally, annual household income does not influence cigarette dependence.

These estimation results suggest that all four hypotheses are supported. The elasticities of smoking probability for these parameters are displayed in Table 5.

*Constant time preference* is positively associated with smoking probability

A 1% increase in the time preference rate significantly increased smoking probability by 0.68%.

*Present bias* is positively associated with smoking probability

A 1% increase in the present bias effect increased smoking probability by 0.42% at the 1% significance level.

**Table 5:** Smoking probabilities elasticities for binomial probit model

	**Elasticity**	**S.E.**	
δ(constant time preference)	0.6764	0.1079	***
1-exp(β) (present bias)	0.4166	0.1543	***

Note 1: ***1% significant level (p < 0.01)

Note 2: The *present bias* elasticities are calculated only for the time-inconsistent samples.

Next, the elasticities of cigarette dependence with respect to the constant time preference and present bias parameters are displayed in Table 6.

*Constant time preference* is proportionally associated with cigarette dependence

A 1% increase in the time preference rate decreased the proportion of low nicotine-dependent smokers by 1.21% at the 1% significance level but increased the proportion of highly nicotine-dependent smokers by 0.84% at the 1% significance level. However, a 1% increase in the time preference rate did not influence the proportion of moderately nicotine-dependent smokers.

*Present bias* is proportionally associated with cigarette dependence

A 1% increase in the present bias effect decreased the proportion of low nicotine-dependent smokers by 0.43% at the 5% significance level but increased the proportion of highly nicotine-dependent smokers by 0.27% at the 5% significance level. However, a 1% increase in the present bias effect did not influence the proportion of moderately nicotine-dependent smokers.

**Table 6:** Cigarette dependence elasticities for ordered probit model

**L-Smoker**			
	Elasticity	S.E.	
δ (constant time preference)	-1.2071	0.4422	***
1-exp(β) (present bias)	-0.4268	0.1905	**
**M-Smoker**			
	Elasticity	S.E.	
δ (constant time preference)	1.0950	0.6759	
1-exp(β) (present bias)	0.5727	0.3818	
**H-Smoker**			
	Elasticity	S.E.	
δ (constant time preference)	0.8404	0.3220	**
1-exp(β) (present bias)	0.2658	0.1266	**

Note 1: ***1% significant level (p < 0.01), **5% significant level (p < 0.05).

Note 2: The *present bias* elasticities are calculated only for the time-inconsistent samples.

## Discussions

Mitchell and Wilson [20] discussed that smokers discount delayed rewards more steeply than non-smokers and found that such discounting is steeper for questions that include an immediate alternative compared with ones in which both rewards are delayed. Moreover, they showed that the heightened discounting of delayed rewards by smokers compared with non-smokers is not confined to situations in which one reward is available immediately. Further, these differences in discounting are driven not only by divergent responses to immediate rewards but also by the inter-reward interval.

Although this paper shares several common aspects with that presented by Mitchell and Wilson [20], there remain some important differences. They compared 20 smokers with 20 non-smokers using a delay discounting task that included Small-Now versus Large-Late and Small-Soon versus Large-Later questions^c^ and employed both real and hypothetical rewards. By contrast, the present paper was rather concerned with hypothetical Small-Now versus Large-Later questions and explicitly measured the relationships between two key parameters (constant time preference and present bias) and two smoking behaviors (smoking and cigarette dependence) according to a quasi-hyperbolic discounting approach using a large sample.

The first conclusion of this paper is that the time preference rate is positively associated with smoking. Because smoking involves considerations such as current stress relief and future health damage, this explains the positive correlation between the time preference rate and smoking probability. Moreover, the finding that smokers are more impatient than non-smokers with regard to delay discounting is consistent with the findings of previous research (Mitchell [16]; Reynolds et al. [17]; Bickel et al. [21]; Odum et al. [22]; Baker et al. [23]; Reynolds et al. [24]). However, this study is unable to determine whether an impatient person tends to smoke or whether a smoker tends to become impatient. A detailed study of causality is therefore the most crucial area for future research.

Second, and most importantly, an individual that has a higher present bias effect tends to be more likely to smoke, because he or she emphasizes present utility (such as temporary stress relief). Consequently, the present bias effect also successfully accounts for smoking decisions. If one supposes that smoking results from discounted utility anomalies, higher consistency naturally leads to lower smoking probability. This result is consistent with the several studies that have regarded addiction as time-inconsistent behavior. For example, Gruber and Koszegi [3] demonstrated that some smokers fail to recognize the true difficulty of quitting. Likewise, Kan [4] empirically studied time-inconsistent preferences in the context of cigarette smoking behavior and concluded that some smokers who want to quit demanded control devices, such as smoking bans in public areas and hikes in cigarette taxes.

Third, there exists a relationship between cigarette dependence and the time preference rate. In other words, a smoker that has a higher time preference rate tends to be more heavily addicted, which is consistent with the findings of previous research. For example, Reynolds et al. [24] reported a significant positive correlation between the number of cigarettes smoked daily and the time preference rate, while Ohmura et al. [18] suggested that both the frequency of nicotine self-administration and the dosage are positively associated with greater delay discounting.

Finally, we showed that a smoker with a higher present bias effect tends to be more heavily addicted. This finding that the present bias effect also successfully accounts for cigarette dependence is also consistent with those of previous research. For example, Gruber and Koszegi [3] developed a new time inconsistency model and argued that government policy should consider not only the externalities that smokers impose on others but also the internalities imposed by smokers on themselves. Similarly, under their concept of *libertarian paternalism,* Thaler and Sunstein [25] insisted that bounded rationality makes it preferable to maintain freedom of choice and design private and public institutions for improving people’s welfare if smokers are unconscious of their inconsistency. Thus, both constant time preference and present bias can suitably account for smoking and cigarette dependence^d^.

## Conclusions

Addiction has attracted considerable attention in health and behavioral economics. This paper investigated smoking, including cigarette dependence, the most common form of addiction, by using the quasi-hyperbolic discounting approach. We found that the higher the time preference rate and the present bias effect, the higher are smoking probability and cigarette dependence. We thus conclude that quasi-hyperbolic discounting parameters function as good predictors of smoking status, which marks a breakthrough in smoking research.

However, some unsolved problems remain. As noted earlier, this research only investigated the relationship between smoking and time preferences and thus a detailed study of causality between impatience and smoking tendency is a crucial area for future research. In addition, the individual-level estimates of *β*-*δ* necessary to identify the time-inconsistent sample in a single task did not stably converge because the preference parameters were qualitatively different between the time-consistent and time-inconsistent samples. Future research should thus aim to integrate them into a one-shot task.

Further, this paper assumed that delay and risk were could be distinguished by the questionnaires. However, some studies, including Rachlin et al. [26], Rachlin and Siegel [27], and Sozou [28], have demonstrated that both risk and the delay of reward can be elicited from the same underlying form of intolerance, because the value of a future reward should be discounted such that there exists a risk that the reward will not be realized. Andersen et al. [29] also argued that allowing for risk preference leads to a significant difference in elicited discount rates. In contrast to these findings, Green and Myerson [30] showed that time and probability discounting are different and dissociable processes. I consider these issues to be potential topics for future research.

## Endnotes

^a^Fehr and Zych [31] reported that addicts systematically consume too much compared with the optimal consumption decision and explained this systematic excess consumption in terms of the psychologically salient features of addictive goods. Additionally, reinforcement matters for addiction, because a larger stock of past consumption raises the marginal utility of current consumption (Becker and Murphy [32]).

^b^Olson and Bailey [33] also illustrate that most analyses of intertemporal choice assumed both diminishing marginal utility (i.e., a concave instantaneous utility function *U*(*C*_*t*_)) and positive time preference (i.e., a positive discount rate *δ*). These two assumptions create opposing forces in intertemporal choice: while diminishing marginal utility motivates a person to spread consumption over time, positive time preference motivates a person to concentrate consumption in the present.

^c^This paper did not fully address the Small-Soon versus Large-Later question. Hence, the *β-δ* framework on decreasing impatience should be developed in future research.

^d^Yuda [34] provides an excellent survey regarding the recent smoking control policies in Japan.

## Competing interests

I have no personal relationship or affiliation (relevant to the subject of the manuscript) that might result in professional gain to a family member.

## Appendix. Generalized RPL model

Assuming that parameter *β*_*n*_ is distributed with density function *f* (*β*_*n*_), the specification allows for repeated choices by each sampled decision maker in such a way that the coefficients vary over people but are constant over choice situations for each person (Train [35]). The logit probability of decision maker *n* choosing alternative *i* in choice situation *t* is expressed as

(15) $$L_{\mathrm{nit}}\left(\beta_{n}\right)=\prod_{t=1}^{T}\left[\exp\left(V_{\mathrm{nit}}\left(\beta_{n}\right)\right)/\sum_{j=1}^{J}\exp\left(V_{\mathrm{njt}}\left(\beta_{n}\right)\right)\right],$$

which is the product of normal logit formulas, given parameter *β*_*n*_, the observable portion of utility function *V*_*nit*_, and alternatives *j = 1, …, J* in choice situations *t = 1, …, T*. Therefore, the choice probability is a weighted average of logit probability *L*_*nit*_ (*β*_*n*_) evaluated at parameter *β*_*n*_ with density function *f* (*β*_*n*_), which can be written as

(16) $$P_{\mathrm{nit}}=\int L_{\mathrm{nit}}\left(\beta_{n}\right)f\left(\beta_{n}\right)d\beta_{n}$$

In the linear-in-parameter form, the utility function can be written as

(17) Unit=βn'xnit+εnit,

where *x*_*nit*_ denotes observable variables, *β*_*n*_ denotes a random parameter vector, and *ε*_*nit*_ denotes an independently and identically distributed extreme value term. Furthermore, in the generalized mixed logit model, we assume

(18) $$\beta_{n}=\sigma_{n}\left(\beta +\eta_{n}\right),$$

(19) $$\sigma_{n}=\exp\left(-\tau^{2}/2+\tau \nu_{n}\right),$$

where the random variable σ_*n*_ captures scale heterogeneity while *η*_*n*_ captures taste heterogeneity; *η*_*n*_ and *ν*_*n*_ are assumed to be normally distributed; and *β* and *τ* are parameters to be estimated.

Since choice probability is not expressed in closed form, simulations need to be performed for the model estimation (see Train [35], p. 148 for details). One can also calculate the estimator of the conditional mean of the random parameters, conditioned on individual specific choice profile *y*_*n*_, given as

(20) $$h\left(\beta_{n}|y_{n}\right)=\left[P\left(y_{n}|\beta_{n}\right)f\left(\beta_{n}\right)\right]/\int P\left(y_{n}|\beta_{n}\right)f\left(\beta_{n}\right)d\beta_{n}.$$

Here, I assume that the preference parameters—*constant time preference, present bias*, and *risk—*follow a normal distribution. I have now employed 200 Halton draws for the estimation. Louviere et al. [36] suggested that 100 replications are normally sufficient for a typical problem involving five alternatives, 1,000 observations, and up to 10 attributes. Bhat [37] also found that 100 Halton draws are more efficient than 1,000 random draws for simulating an model. Thus, 200 Halton draws seem to be sufficient to obtain stable results.

## References

[B1] Chaloupka FJ Warner KE Newhouse J, Culyer A The economics of smoking The handbook of health economics 1B 2000 Elsevier, Amsterdam: North-Holland 1539 1612 10.1016/S1574-0064(00)80042-6

[B2] Laibson D Golden eggs and hyperbolic discounting Q J Econ 1997 4 443 477 10.1162/003355397555253

[B3] Gruber J Koszegi B Is addiction rational: theory and evidence Q J Econ 2001 4 1261 1303 10.1162/003355301753265570

[B4] Kan K Cigarette smoking and self-control J Health Econ 2007 4 61 81 10.1016/j.jhealeco.2006.07.002 16950529

[B5] Doyle JR Survey of time preference, delay discounting models Judgm Decis Mak 2013 4 116 135

[B6] Van de Ven J Weale M An empirical investigation of quasi-hyperbolic discounting. NIESR discussion papers 355 2010 National Institute of Economic and Social Research, London, United Kingdom

[B7] Fagerström K Determinants of tobacco Use and renaming the FTND to the fagerström test for cigarette dependence Nicotine Tob Res 2011 4 75 78 10.1093/ntr/ntr137 22025545

[B8] Heatherton TF Kozlowski LT Frecker RC Fagerström KO The fagerström test for nicotine dependence: a revision of the fagerström tolerance questionnaire Br J Addict 1991 4 1119 1127 10.1111/j.1360-0443.1991.tb01879.x 1932883

[B9] Frederick S Lowenstein G O’Donoghue T Time discounting and time preference: a critical review J Econ Lit 2002 4 351 401 10.1257/jel.40.2.351

[B10] Strotz R Myopia and inconsistency in dynamic utility maximization Rev Econ Stud 1956 4 165 180 10.2307/2295722

[B11] Prelec D Decreasing impatience: a criterion for Non-stationary time preference and “hyperbolic” discounting Scand J Econ 2004 4 511 532 10.1111/j.0347-0520.2004.00375.x

[B12] Takahashi T A neuroeconomic theory of rational addiction and nonlinear time-perception 2011 21712792

[B13] Ida T Goto R Simultaneous measurement of time and risk preferences: stated preference discrete choice modeling analysis depending on smoking behavior Int Econ Rev 2009 4 1169 1182 10.1111/j.1468-2354.2009.00564.x

[B14] Ida T Anomaly, impulsivity, and addiction J Socio Econ 2010 4 194 203 10.1016/j.socec.2009.10.005

[B15] Fiebig DG Keane MP Louviere J Wasi N The generalized multinomial logit model: accounting for scale and coefficient heterogeneity Mark Sci 2010 4 393 421 10.1287/mksc.1090.0508

[B16] Mitchell SH Measures of impulsivity in cigarette smokers and non-smokers Psychopharmacology 1999 4 455 464 10.1007/PL00005491 10550496

[B17] Reynolds B Karraker K Horn K Richards JB Delay and probability discounting as related to different stages of adolescent smoking and Non-smoking Behav Process 2003 4 333 344 10.1016/S0376-6357(03)00168-2 14580702

[B18] Ohmura Y Takahashi T Kitamura N Discounting delayed and probabilistic monetary gains and losses by smokers of cigarettes Psychopharmacology 2005 4 508 515 10.1007/s00213-005-0110-8 16167142

[B19] McKelvey RD Zavoina W A statistical model for the analysis of ordinal level dependent variables J Math Sociol 1975 4 103 120 10.1080/0022250X.1975.9989847

[B20] Mitchell SH Wilson VB Differences in delay discounting between smokers and nonsmokers remain when both rewards are delayed Psychopharmacology 2012 4 549 562 10.1007/s00213-011-2521-z 21983917 PMC3677053

[B21] Bickel WK Odum AL Madden GJ Impulsivity and cigarette smoking: delay discounting in current never, and ex-smokers Psychopharmacology 1999 4 447 454 10.1007/PL00005490 10550495

[B22] Odum AL Madden GJ Bickel WK Discounting of delayed health gains and losses by current, never- and ex-smokers of cigarettes Nicotine Tob Res 2002 4 295 303 10.1080/14622200210141257 12215238

[B23] Baker F Johnson MW Bickel WK Delay discounting in current and never-before cigarette smokers: similarities and differences across commodity, sign, and magnitude J Abnorm Psychol 2003 4 382 392 10.1037/0021-843X.112.3.382 12943017

[B24] Reynolds B Richards JB Horn K Karraker K Delay discounting and probability discounting as related to cigarette smoking status in adults Behav Process 2004 4 35 42 10.1016/S0376-6357(03)00109-8 14744545

[B25] Thaler RH Sunstein CR Nudge: improving decisions about health, wealth, and happiness 2008 Yale University Press, New Haven: United States

[B26] Rachlin H Raineri A Cross D Subjective probability and delay J Exp Anal Behav 1991 4 233 244 10.1901/jeab.1991.55-233 2037827 PMC1323057

[B27] Rachlin H Siegel E Temporal pattering in probabilistic choice Organ Behav Hum Decis Process 1994 4 161 176 10.1006/obhd.1994.1054

[B28] Sozou PD On hyperbolic discounting and uncertain hazard rates Proceedings of the Royal Soc B: Biol Sci 1998 4 2015 2020 10.1098/rspb.1998.0534

[B29] Andersen S Harrison GW Lau MI Rutström EE Eliciting risk and time preferences Econometrica 2008 4 583 618 10.1111/j.1468-0262.2008.00848.x

[B30] Green L Myerson J A discounting framework for choice with delayed and probabilistic rewards Psychol Bull 2004 4 769 792 10.1037/0033-2909.130.5.769 15367080 PMC1382186

[B31] Fehr E Zych PK Do addicts behave rationally? Scand J Econ 1998 4 643 661 10.1111/1467-9442.00127

[B32] Becker GS Murphy KM A theory of rational addiction J Polit Econ 1988 4 675 700 10.1086/261558

[B33] Olson M Bailey MJ Positive time preference J Polit Econ 1981 4 1 25 10.1086/260947

[B34] Yuda M The impacts of recent smoking control policies on individual smoking choice: the case of Japan Heal Econ Rev 2013 4 4 1 13 10.1186/2191-1991-3-4 PMC3606835 23497490

[B35] Train KE Discrete choice methods with simulation 2003 Cambridge University Press, Cambridge: United Kingdom

[B36] Louviere JJ Hensher DA Swait JD Stated choice methods 2000 Cambridge University Press, Cambridge

[B37] Bhat C Quasi-random maximum simulated likelihood estimation of the mixed multinomial logit model Transport Res 2001 4 677 693 10.1016/S0191-2615(00)00014-X

